# The global burden and attributable risk factor analysis of acute myeloid leukemia in 195 countries and territories from 1990 to 2017: estimates based on the global burden of disease study 2017

**DOI:** 10.1186/s13045-020-00908-z

**Published:** 2020-06-08

**Authors:** Ming Yi, Anping Li, Linghui Zhou, Qian Chu, Yongping Song, Kongming Wu

**Affiliations:** 1 Department of Oncology, Tongji Hospital of Tongji Medical College, Huazhong University of Science and Technology, Wuhan, 430030 China; 2 The Affiliated Cancer Hospital of Zhengzhou University & Henan Cancer Hospital, Zhengzhou, 450008 China; 3 Department of Oncology, The Second Affiliated Hospital of Xi’an Jiaotong University, Xi’an, 710004 China

**Keywords:** Acute myeloid leukemia, Global burden disease, Cancer epidemiology, Cancer statistics, Population aging

## Abstract

**Background:**

Acute myeloid leukemia (AML) is a common leukemia subtype and has a poor prognosis. The risk of AML is highly related to age. In the context of population aging, a comprehensive report presenting epidemiological trends of AML is evaluable for policy-marker to allocate healthy resources.

**Methods:**

This study was based on the Global Burden of Disease 2017 database. We analyzed the change trends of incidence rate, death rate, and disability-adjusted life year (DALY) rate by calculating the corresponding estimated annual percentage change (EAPC) values. Besides, we investigated the influence of social development degree on AML’s epidemiological trends and potential risk factors for AML-related mortality.

**Results:**

From 1990 to 2017, the incidence of AML gradually increased in the globe. Males and elder people had a higher possibility to develop AML. Developed countries tended to have higher age-standardized incidence rate and death rate than developing regions. Smoking, high body mass index, occupational exposure to benzene, and formaldehyde were the main risk factors for AML-related mortality. Notably, the contribution ratio of exposure to carcinogens was significantly increased in the low social-demographic index (SDI) region than in the high SDI region.

**Conclusion:**

Generally, the burden of AML became heavier during the past 28 years which might need more health resources to resolve this population aging-associated problem. In the present stage, developed countries with high SDI had the most AML incidences and deaths. At the same time, developing countries with middle- or low-middle SDI also need to take actions to relieve rapidly increased AML burden.

## Background

Acute myeloid leukemia (AML) is a malignant disease of hemopoietic stem cells or progenitors which is characterized as the differentiation arrest and aberrant proliferation of myeloid lineages [1]. In 2017, AML is the most commonly diagnosed acute leukemia subtype in the USA [2]. AML could occur in any age group especially in elder individuals. It was reported that nearly 75% of AML patients were aged 65 years or older in the USA [3]. The mechanisms by which AML develops have not been completely understood. It is generally believed that the oncogenic transformation of hemopoietic stem cells or progenitors initiates leukemogenesis [4]. These specific mutations in the early stage of leukemogenesis bring a selective advantage for hemopoietic stem cells or progenitors during clonal expansion, which might impair normal hemopoiesis and eventually develop into AML [5]. The alterations of genes such as *FLT3*, *IDH1*, *IDH2*, *TET2*, *ASXL1*, and *DNMT3A* could be found in the bone marrow or peripheral blood of patients without overt AML [6–12]. This status is termed clonal hematopoiesis of indeterminate potential (Chip) [13]. For patients with Chip, the rate of transformation to overt hematologic malignancy is about 0.5–1% per year [14]. It is notable that approximately 10% AML patients underwent cytotoxic chemotherapy or radiotherapy previously, usually as the treatment for primary tumor [15]. For patients harboring Chip, the risk of having AML is increased after cytotoxic treatment [5]. Some somatic mutations such as *TP53* mutation endow preleukemic hemopoietic stem cells with enhanced resistance to chemotherapy which further elevates the competitive advantage over normal hemopoietic stem cells [16, 17].

According to SEER database, over ten thousand people died from AML which accounted for 62% of all leukemia-related deaths in the USA [3]. In the present stage, the median survival time of AML is nearly 8.5 months [3]. The 2-year and 5-year overall survival (OS) rates are 32% and 24% [3]. With several recent drug approvals for precision therapy of AML, significant progress has been made in improving the outcomes of AML [18–25]. In addition, this improvement in AML’s outcomes is also partly attributed to better supportive care such as more effective antimicrobials [26]. Age at diagnosis is an important factor determining the long-term survival of AML patients. It was reported that the 2-year and 5-year OS rates of individuals diagnosed before the age of 40 were five-fold higher than patients diagnosed at 65 years or older [27]. Besides, patients’ lifestyle such as smoking and sociodemographic factor also have impacts on AML patients’ survival [28–30]. Epidemiological investigations of AML are valuable references for policy-makers to allocate healthy resources. In this study, we presented in detail the statistical data of AML in the globe, different regions, and 195 countries or territories from 1999 to 2017. Moreover, we tried to analyze the influence of multiple risk factors on AML-related mortality.

## Methods

### Data acquisition and download

The Global Burden of Disease (GBD) database contains the statistical data of 354 diseases in 195 countries or territories [31, 32]. The data of AML including incidences, death cases, disability-adjusted life years (DALYs), and corresponding age-standardized rates were downloaded from the Global Health Data Exchange (GHDx) website (http://ghdx.healthdata.org/gbd-results-tool). The background information such as social demographic index (SDI) was also downloaded for the following correlation analysis. SDI values range between 0 and 1 which reflect the degree of social development.

### Statistical analysis

Annual incidence cases, deaths, DALYs, and corresponding age-standardized rates (ASRs) were used to describe the burden to AML. ASRs could exclude the interference from changes in age distribution and population quantity. DALY was the summation of the years lived with disability and the years of life lost. Moreover, EAPC based on ASRs including age-standardized incidence/death rate (ASIR/ASDR) and age-standardized DALY rate per 100,000 persons were employed to reflect the change trends of AML’s burden. In the formula *y = α + βx*, *y* means log_10_ (ASR) value while *x* refers to the calendar year. EAPC values were calculated based on the formula EAPC = 100* (10^β -1). For EAPC value and its 95% confidence interval (CI) above zero, the corresponding ASR was in an upward trend and vice versa. To investigate the correlation between ASR change trends and social development degrees, we calculated Pearson’s correlation coefficient between EAPCs and SDI values. Lastly, we searched the GBD database for potential risk factors contributing to AML-related mortality and visualized corresponding results.

### Data visualization

All data analysis was based on the open-source software R (version 3.6.0). The data visualization was performed with packages including maps, ggplot2, and RcolorBrewer. Data cleaning was conducted with package dplyr. Histograms were used to demonstrate the quantities and change trends of AML’s incidence cases, deaths, and DALYs from 1990 to 2017. Maps were adopted for the visual presentation of AML’s burden and corresponding ASRs in the 195 countries and territories. Scatter diagrams and regression curves were employed to analyze the correlation between ASRs and SDI values. Area under the curve was used to present the dynamic distribution of the age composition of AML patients.

## Results

### The incidence and its change trend of AML

**Fig. 1:**
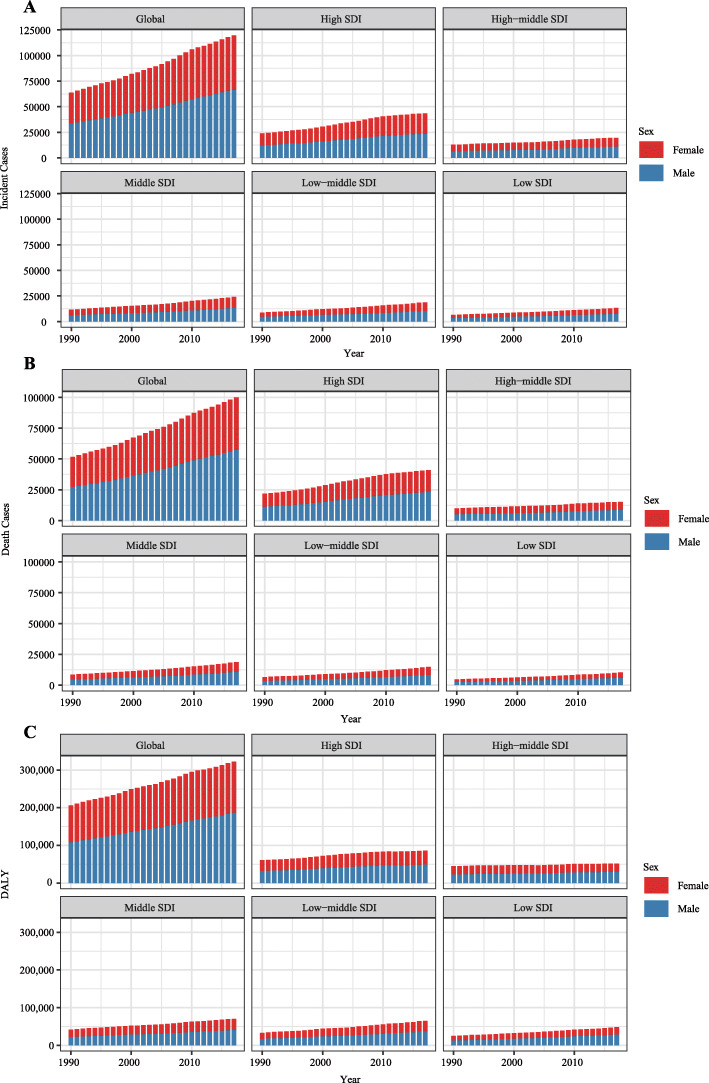
The change trends of AML’s incidence cases, deaths, and DALYs from 1990 to 2017. **a** The change trends of incidences, **b** the change trends of deaths, and **c** the change trends of DALYs. Blue bars represent males and red bars represent females. Note: AML, acute myeloid leukemia; DALY, disability-adjusted life year

**Fig. 2:**
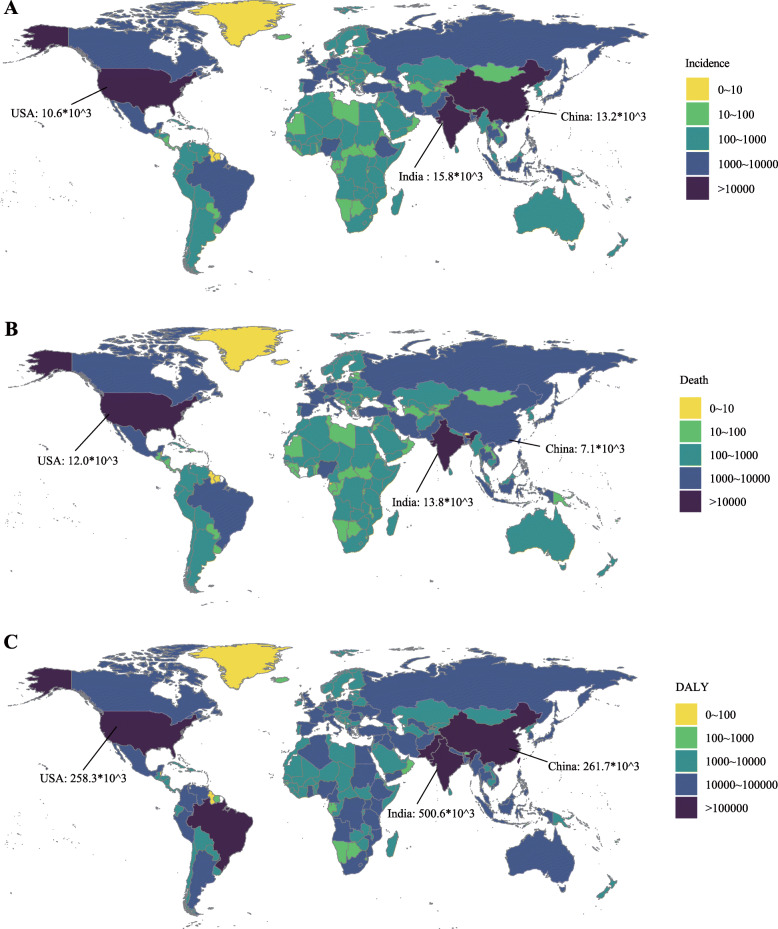
The global disease burden of AML in 195 countries or territories. **a** The incidence cases of 195 countries or territories in 2017, **b** the deaths of 195 countries or territories in 2017, and **c** the DALYs of 195 countries or territories in 2017. Note: AML, acute myeloid leukemia; DALY, disability-adjusted life year

**Fig. 3:**
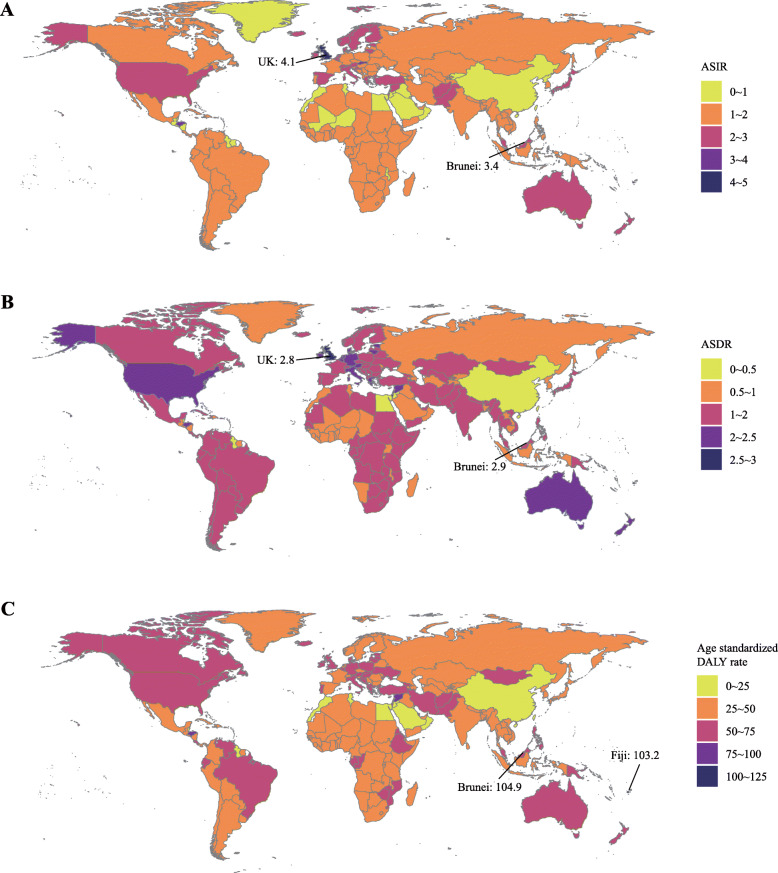
The age-standardized rates of AML in 195 countries or territories. **a** The ASIR of 195 countries or territories in 2017, **b** the ASDR of 195 countries or territories in 2017, and **c** the age-standardized DALY rate of 195 countries or territories in 2017. Note: AML, acute myeloid leukemia; ASIR, age-standardized incidence rate; ASDR, age-standardized death rate; DALY, disability-adjusted life year

In the globe, the incidence case of AML was increased gradually in the past 28 years (from 63.84 × 10^3^ in 1990 to 119.57 × 10^3^ cases in 2017, increasing by 87.3%, EAPC = 0.56, 95% CI 0.49~0.62) (Table 1) (Fig. 1a). Males were more likely to suffer from AML than females (male to female ratio in ASIR = 1.23:1 in 1990, and 1.38:1 in 2017). In the region level, the high SDI region had the highest AML burden until 2017 (incidence case: 23.86 × 10^3^ in 1990 and 43.42 × 10^3^ cases in 2017). In the meanwhile, middle SDI had the most rapid increase during the 28 years (ASIR: 0.86 in 1990 and 1.14 in 2017, EAPC = 1.03, 95% CI 1.00~1.07). Subgroup analysis by geographical zone showed Western Europe and South Asia had the most incidence cases (Western Europe: 11.94 × 10^3^ in 1990 and 20.02 × 10^3^ in 2017; South Asia: 10.06 × 10^3^ in 1990 and 21.46 × 10^3^ in 2017). Andean Latin America and East Asia had the most mushrooming rise (EAPC of Andean Latin America: 1.68, 95% CI 1.55~1.82; EAPC of East Asia: 1.55, 95% CI 1.40~1.69). In the country or territory level, India, China, and the USA had the most incidence cases (7.4 × 10^3^, 6.9 × 10^3^, and 5.4 × 10^3^ cases in 1990, respectively; 15.8 × 10^3^, 13.2 × 10^3^, 10.6 × 10^3^ cases in 2017, respectively) (Fig. 2a) (Additional file 1:Table S1 and Table S7). The UK had the highest ASIR both in 1990 and 2017 (ASIR = 4.19 in 1990 and 4.05 in 2017) (Fig. 3a) (Additional file 1: Table S4 and Table S10). Ecuador had the most prompt increase in ASIR (EAPC = 3.31, 95% CI 2.94~3.69) (Additional file 1: Table S13).

**Table 1:** The incidence of AML in 1990/2017 and temporal trends

	1990		2017		1990–2017
	Incident cases No *10^**3**^ (95% CI)	ASIR/100,000 No. (95% CI)	Incident cases No *10^**3**^ (95% CI)	ASIR/100,000 No. (95% CI)	EAPC No. (95% CI)
**Overall**	63.84 (54.95~83.84)	1.35 (1.20~1.71)	119.57 (108.37~125.93)	1.54 (1.40~1.63)	0.56 (0.49~0.62)
**Sex**					
**Male**	33.60 (29.51~40.47)	1.51 (1.36~1.74)	66.79 (57.83~71.62)	1.81 (1.57~1.94)	0.72 (0.67~0.77)
**Female**	30.24 (24.37~45.16)	1.23 (1.02~1.77)	52.79 (46.07~57.78)	1.31 (1.14~1.44)	0.35 (0.27~0.44)
**Socio-demographic factor**					
**High SDI**	23.86 (23.01~24.9)	2.05 (1.97~2.15)	43.42 (41.11~45.61)	2.29 (2.18~2.41)	0.60 (0.45~0.74)
**High-middle SDI**	12.86 (11.22~15.81)	1.21 (1.06~1.47)	19.63 (17.05~21.18)	1.30 (1.12~1.41)	0.36 (0.29~0.43)
**Middle SDI**	11.58 (9.55~15.89)	0.86 (0.73~1.13)	24.05 (20.77~26.25)	1.14 (0.98~1.24)	1.03 (1.00~1.07)
**Low-middle SDI**	8.81 (6.43~13.60)	1.01 (0.79~1.42)	18.82 (16.53~21.75)	1.27 (1.12~1.47)	0.82 (0.77~0.88)
**Low SDI**	6.55 (3.83~13.98)	1.16 (0.80~1.99)	13.34 (10.84~15.41)	1.31 (1.07~1.48)	0.34 (0.29~0.38)
**Region**					
**Andean Latin America**	0.32 (0.24~0.44)	0.97 (0.78~1.27)	0.82 (0.59~0.97)	1.40 (1.02~1.65)	1.68 (1.55~1.82)
**Australasia**	0.66 (0.59~0.76)	2.92 (2.59~3.29)	1.10 (0.90~1.31)	2.63 (2.17~3.13)	− 0.74 (− 0.99~− 0.48)
**Caribbean**	0.42 (0.34~0.62)	1.30 (1.07~1.82)	0.61 (0.53~0.74)	1.28 (1.10~1.56)	0.02 (− 0.13~0.17)
**Central Asia**	0.78 (0.67~0.95)	1.17 (1.03~1.39)	1.13 (1.00~1.26)	1.28 (1.15~1.43)	0.63 (0.50~0.76)
**Central Europe**	2.07 (1.90~2.27)	1.52 (1.38~1.67)	2.56 (2.29~2.73)	1.56 (1.39~1.71)	0.48 (0.36~0.60)
**Central Latin America**	1.64 (1.55~1.89)	1.16 (1.11~1.29)	3.41 (3.20~3.65)	1.38 (1.29~1.47)	0.63 (0.54~0.72)
**Central Sub-Saharan Africa**	0.52 (0.31~1.05)	1.25 (0.86~1.73)	1.14 (0.80~1.61)	1.29 (0.89~1.59)	− 0.05 (− 0.16~0.07)
**East Asia**	7.61 (5.56~11.79)	0.64 (0.48~0.97)	14.79 (11.96~16.70)	0.95 (0.77~1.08)	1.55 (1.40~1.69)
**Eastern Europe**	3.76 (3.06~4.28)	1.59 (1.30~1.87)	3.59 (3.20~4.06)	1.39 (1.21~1.62)	− 0.59 (− 0.75~− 0.43)
**Eastern sub-Saharan Africa**	1.64 (1.06~2.77)	1.13 (0.85~1.63)	4.01 (2.95~4.95)	1.38 (1.06~1.67)	0.62 (0.53~0.71)
**High-income Asia Pacific**	3.51 (3.29~3.82)	1.87 (1.74~2.06)	7.38 (6.45~8.38)	2.09 (1.83~2.37)	0.74 (0.48~1.01)
**High-income North America**	5.95 (5.75~6.23)	1.78 (1.72~1.87)	11.70 (11.16~12.30)	2.22 (2.11~2.35)	1.04 (0.78~1.29)
**North Africa and Middle East**	4.17 (2.83~6.68)	1.48 (1.03~2.15)	6.99 (5.80~8.41)	1.30 (1.08~1.55)	− 0.32 (− 0.43~− 0.22)
**Oceania**	0.09 (0.06~0.14)	1.75 (1.31~2.47)	0.19 (0.14~0.29)	1.81 (1.38~2.52)	0.23 (0.16~0.30)
**South Asia**	10.06 (6.84~17.32)	1.14 (0.86~1.70)	21.46 (18.64~24.64)	1.40 (1.22~1.60)	0.66 (0.59~0.74)
**Southeast Asia**	3.76 (2.68~6.34)	1.01 (0.77~1.59)	8.97 (7.48~10.39)	1.43 (1.19~1.64)	1.38 (1.29~1.48)
**Southern Latin America**	0.69 (0.63~0.78)	1.43 (1.29~1.60)	1.09 (0.98~1.22)	1.49 (1.33~1.69)	0.18 (0.08~0.27)
**Southern sub-Saharan Africa**	0.55 (0.41~0.64)	1.37 (1.01~1.64)	0.92 (0.68~1.08)	1.37 (1.02~1.60)	− 0.26 (− 0.56~0.05)
**Tropical Latin America**	2.04 (1.90~2.18)	1.56 (1.47~1.64)	3.59 (3.38~3.77)	1.60 (1.49~1.68)	0.11 (0.02~0.21)
**Western Europe**	11.94 (11.36~12.75)	2.32 (2.20~2.46)	20.02 (18.46~21.51)	2.50 (2.30~2.70)	0.40 (0.32~0.48)
**Western sub-Saharan Africa**	1.63 (1.06~2.37)	0.92 (0.67~1.17)	4.12 (2.93~5.05)	1.07 (0.78~1.32)	0.44 (0.34~0.54)

Note: *ASIR* age-standardized incidence rate

### The death and its change trend of AML

Generally, AML-related death was remarkably increased from 51.77 × 10^3^ cases in 1990 to 99.90 × 10^3^ cases in 2017 (increasing by 93.0%, EAPC = 0.45, 95% CI 0.36~0.53) (Table 2) (Fig. 1b). The number of AML-related death in males was higher than in females (1990: 27.33 × 10^3^ cases in male and 24.43 × 10^3^ cases in females; 2017: 57.40 × 10^3^ cases in males and 42.50 × 10^3^ cases in females). Subgroup analysis by a socio-demographic factor indicated that the high SDI region had the most death cases (21.87 × 10^3^ death cases in 1990 and 40.91 × 10^3^ death cases in 2017). At the same time, the middle SDI and low-middle SDI regions had a relatively huge rise in ASDR (EAPC of middle SDI: 0.85, 95% CI 0.83~0.87; EAPC of low-middle SDI: 0.93, 95% CI 0.89~0.98). As for a specific geographical zone, Western Europe, South Asia, and high-income North America zones were the top 3 regions with the most AML-related deaths (Western Europe: 9.81 × 10^3^ death cases in 1990 and 18.22 × 10^3^ death cases in 2017; South Asia: 7.60 × 10^3^ death cases in 1990 and 18.07 × 10^3^ death cases in 2017; high-income North America: 6.98 × 10^3^ death cases in 1990 and 13.21 × 10^3^ death cases in 2017). Andean Latin America had the fastest rise in ASDR (EAPC = 1.87, 95% CI 1.73~2.01). Subgroup analysis by country or territory showed that the USA, India, and China were the top 3 countries with the most death cases (1990: 6.4 × 10^3^, 5.7 × 10^3^, and 4.5 × 10^3^, respectively; 2017: 12.0 × 10^3^, 13.8 × 10^3^, and 7.1 × 10^3^, respectively) (Fig. 2b) (Additional file 1: Table S2 and Table S8). The UK had almost the highest ASDR in 1990 and 2017 (ASDR = 2.9 in 1990, ranking second; ASDR = 2.8 in 2017, ranking second) (Fig. 3b) (Additional file 1: Table S5 and Table S11). El Salvador and Ecuador had the most rapid increase in ASDR (El Salvador: EAPC = 3.62, 95% CI 2.93~4.31; Ecuador: EAPC = 3.53, 95% CI 3.13~3.93) (Additional file 1: Table S14).

**Table 2:** The death of AML in 1990/2017 and temporal trends

	1990		2017		1990–2017
	Death cases No *10^**3**^ (95% CI)	ASDR/100,000 No. (95% CI)	Death cases No *10^**3**^ (95% CI)	ASDR/100,000 No. (95% CI)	EAPC No. (95% CI)
**Overall**	51.77 (46.26~64.34)	1.16 (1.06~1.39)	99.90 (91.28~104.58)	1.28 (1.17~1.34)	0.45 (0.36~0.53)
**Sex**					
**Male**	27.33 (24.67~31.31)	1.33 (1.21~1.48)	57.40 (50.85~60.98)	1.58 (1.40~1.67)	0.74 (0.66~0.83)
**Female**	24.43 (20.60~34.16)	1.04 (0.89~1.40)	42.50 (37.88~46.22)	1.03 (0.92~1.13)	0.06 (− 0.04~0.15)
**Socio-demographic factor**					
**High SDI**	21.87 (21.39~22.78)	1.80 (1.76~1.86)	40.91 (39.11~42.19)	1.96 (1.88~2.03)	0.48 (0.32~0.65)
**High-middle SDI**	9.98 (9.03~11.69)	0.96 (0.88~1.12)	15.11 (13.55~15.94)	0.93 (0.83~0.98)	− 0.08 (− 0.13~− 0.02)
**Middle SDI**	8.54 (7.29~11.17)	0.69 (0.61~0.88)	18.64 (16.05~20.01)	0.87 (0.75~0.93)	0.85 (0.83~0.87)
**Low-middle SDI**	6.51 (4.96~9.58)	0.84 (0.68~1.11)	14.77 (13.06~17.11)	1.08 (0.96~1.24)	0.93 (0.89~0.98)
**Low SDI**	4.75 (3.00~9.30)	0.98 (0.72~1.51)	10.28 (8.34~11.64)	1.14 (0.94~1.28)	0.49 (0.46~0.53)
**Region**					
**Andean Latin America**	0.23 (0.18~0.31)	0.79 (0.65~0.99)	0.67 (0.50~0.77)	1.19 (0.88~1.35)	1.87 (1.73~2.01)
**Australasia**	0.64 (0.60~0.67)	2.72 (2.58~2.83)	1.10 (0.98~1.21)	2.39 (2.16~2.62)	− 0.87 (− 1.1~− 0.65)
**Caribbean**	0.34 (0.29~0.46)	1.11 (0.96~1.44)	0.54 (0.48~0.64)	1.11 (0.97~1.32)	0.11 (− 0.06~0.28)
**Central Asia**	0.58 (0.52~0.68)	0.93 (0.84~1.08)	0.89 (0.82~0.96)	1.05 (0.97~1.14)	0.79 (0.64~0.95)
**Central Europe**	2.03 (1.91~2.20)	1.42 (1.33~1.55)	3.32 (3.02~3.48)	1.73 (1.58~1.81)	1.12 (0.98~1.26)
**Central Latin America**	1.21 (1.17~1.34)	0.95 (0.93~1.02)	2.78 (2.59~2.91)	1.14 (1.06~1.19)	0.69 (0.61~0.77)
**Central sub-Saharan Africa**	0.38 (0.25~0.67)	1.10 (0.76~1.39)	0.86 (0.60~1.07)	1.14 (0.78~1.45)	0.01 (− 0.13~0.15)
**East Asia**	4.92 (3.73~7.32)	0.44 (0.34~0.63)	7.91 (6.53~8.75)	0.46 (0.38~0.51)	0.09 (− 0.04~0.22)
**Eastern Europe**	3.13 (2.59~3.39)	1.23 (1.01~1.31)	3.23 (2.99~3.40)	1.09 (1.02~1.16)	− 0.49 (− 0.60~− 0.38)
**Eastern sub-Saharan Africa**	1.17 (0.80~1.85)	0.99 (0.77~1.36)	2.92 (2.19~3.53)	1.22 (0.95~1.45)	0.70 (0.63~0.77)
**High-income Asia Pacific**	2.98 (2.90~3.07)	1.53 (1.49~1.58)	5.74 (5.44~6.02)	1.44 (1.35~1.53)	0.05 (− 0.17~0.27)
**High-income North America**	6.98 (6.84~7.25)	2.00 (1.96~2.08)	13.21 (12.76~13.62)	2.26 (2.19~2.34)	0.50 (0.31~0.68)
**North Africa and Middle East**	3.07 (2.04~4.75)	1.22 (0.82~1.70)	5.53 (4.56~6.57)	1.11 (0.92~1.31)	− 0.14 (− 0.25~− 0.04)
**Oceania**	0.06 (0.05~0.10)	1.43 (1.06~1.86)	0.14 (0.10~0.19)	1.47 (1.12~1.93)	0.25 (0.15~0.35)
**South Asia**	7.60 (5.45~12.07)	0.98 (0.76~1.36)	18.07 (15.70~20.54)	1.25 (1.08~1.42)	0.84 (0.77~0.91)
**Southeast Asia**	2.83 (2.13~4.58)	0.84 (0.67~1.27)	6.83 (5.60~8.01)	1.12 (0.91~1.30)	1.22 (1.11~1.34)
**Southern Latin America**	0.60 (0.55~0.65)	1.24 (1.15~1.34)	1.02 (0.93~1.11)	1.32 (1.21~1.45)	0.29 (0.17~0.41)
**Southern sub-Saharan Africa**	0.44 (0.33~0.53)	1.21 (0.87~1.48)	0.77 (0.57~0.89)	1.21 (0.91~1.40)	− 0.22 (− 0.48~0.04)
**Tropical Latin America**	1.60 (1.53~1.67)	1.33 (1.28~1.38)	3.27 (3.06~3.39)	1.44 (1.35~1.49)	0.32 (0.21~0.43)
**Western Europe**	9.81 (9.47~10.55)	1.79 (1.73~1.90)	18.22 (17.16~19.16)	2.13 (2.00~2.25)	0.84 (0.65~1.04)
**Western sub-Saharan Africa**	1.14 (0.79~1.56)	0.74 (0.56~0.91)	2.88 (2.02~3.52)	0.87 (0.62~1.09)	0.57 (0.48~0.66)

Note: *ASDR* age-standardized death rate

### The DALY and its change trend of AML

In the globe, the DALY was increased from 2063.15 × 10^3^ in 1990 to 3221.47 × 10^3^ in 2017 (Table 3) (Fig. 1c). Compared with female, male population was the main contributor to the rapidly elevated DALY (male: 1088.68 in 1990 and 1868.89 in 2017, EAPC = 0.46, 95% CI 0.41~0.51; female: 974.48 in 1990 and 1352.58 in 2017, EAPC = − 0.31, 95% CI − 0.37~− 0.24). Subgroup analysis by socio-demographic factor demonstrated that although the high SDI region had the highest DALY from 1990 to 2017 (610.21 × 10^3^ in 1990 and 861.46 × 10^3^ in 2017), the age-standardized DALY rate in the high SDI region was modestly decreased in the past 28 years. In the contrast, the age-standardized DALY rate in the middle SDI and low-middle SDI regions were gradually increased (middle SDI: EAPC = 0.61, 95% CI 0.58~0.65; low-middle SDI: EAPC = 0.74, 95% CI 0.69~0.79). In subgroup analysis by geographical zone, we found that South Asia had the highest DALY until 2017 (369.72 × 10^3^ in 1990 and 705.85 × 10^3^ in 2017). Andean Latin America had the most sharply increase in age-standardized DALY rate (EAPC = 1.59, 95% CI 1.46~1.73). In the level of country or territory, India, China, and the USA had the most DALY (272 × 10^3^, 235 × 10^3^, and 167 × 10^3^ in 1990, respectively; 501 × 10^3^, 262 × 10^3^, and 258 × 10^3^ in 2017, respectively) (Fig. 2c) (Additional file 1: Table S3 and Table S9). Brunei had nearly the highest age-standardized DALY rate from 1990 to 2017 (81 in 1990, ranking sixth; 105 in 2017, ranking first) (Fig. 3c) (Additional file 1: Tables S6 and S12). Ecuador had the fastest increase in age standard DALY rate during the past 28 years (EAPC = 3.19, 95% CI 2.81~3.57) (Additional file 1: Table S15).

**Table 3:** The DALY of AML in 1990/2017 and temporal trends

	1990		2017		1990–2017
	DALY No *10^**3**^ (95% CI)	Age-standardized DALY rate/100,000 No. (95% CI)	DALY No *10^**3**^ (95% CI)	Age-standardized DALY rate/100,000 No. (95% CI)	EAPC No. (95% CI)
**Overall**	2063.15 (1709.81~2905.02)	40.44 (34.40~54.66)	3221.47 (2890.15~3438.43)	41.67 (37.33~44.54)	0.13 (0.07~0.18)
**Sex**					
**Male**	1088.68 (933.08~1391.83)	43.73 (38.41~53.39)	1868.89 (1585.26~2016.44)	49.20 (41.72~53.11)	0.46 (0.41~0.51)
**Female**	974.48 (746.51~1580.45)	37.59 (29.52~59.04)	1352.58 (1179.10~1523.70)	34.54 (30.02~39.09)	− 0.31 (− 0.37~− 0.24)
**Socio-demographic factor**					
**High SDI**	610.21 (593.61~628.16)	56.05 (54.22~57.91)	861.46 (826.28~888.95)	51.97 (49.74~53.94)	− 0.20 (− 0.34~− 0.06)
**High-middle SDI**	447.39 (390.99~561.34)	40.80 (35.79~51.00)	515.17 (458.32~545.11)	34.99 (31.06~37.15)	− 0.55 (− 0.62~− 0.49)
**Middle SDI**	419.96 (338.90~589.84)	28.15 (23.30~38.17)	704.89 (616.44~764.59)	33.32 (29.19~36.24)	0.61 (0.58~0.65)
**Low-middle SDI**	329.85 (230.63~545.05)	32.91 (24.57~50.01)	650.74 (568.11~755.33)	40.40 (35.56~47.05)	0.74 (0.69~0.79)
**Low SDI**	250.16 (133.49~575.40)	37.25 (23.51~72.49)	482.40 (384.94~561.43)	41.21 (33.14~46.98)	0.30 (0.26~0.33)
**Region**					
**Andean Latin America**	11.75 (8.62~16.79)	31.52 (24.54~42.87)	26.54 (19.08~31.12)	44.19 (31.90~51.59)	1.59 (1.46~1.73)
**Australasia**	17.46 (16.64~18.20)	78.76 (74.99~82.18)	23.80 (21.54~26.17)	62.73 (56.85~69.03)	− 1.18 (− 1.37~− 0.98)
**Caribbean**	15.52 (12.25~24.46)	45.23 (36.60~68.30)	20.14 (17.34~24.90)	42.78 (36.63~53.61)	− 0.12 (− 0.28~0.04)
**Central Asia**	30.95 (26.26~37.11)	44.07 (38.45~52.24)	40.44 (36.85~44.28)	44.91 (40.95~49.13)	0.38 (0.25~0.51)
**Central Europe**	68.30 (63.12~74.47)	51.31 (46.80~56.44)	83.31 (76.99~87.51)	52.34 (48.18~55.40)	0.39 (0.27~0.51)
**Central Latin America**	63.33 (60.51~72.47)	39.86 (38.41~44.41)	112.92 (106.23~118.37)	44.64 (41.90~46.78)	0.41 (0.32~0.50)
**Central sub-Saharan Africa**	20.60 (11.43~45.70)	39.92 (25.87~64.43)	44.34 (31.22~66.31)	41.07 (28.80~50.97)	− 0.01 (− 0.14~0.12)
**East Asia**	255.31 (182.27~403.90)	20.44 (14.74~31.95)	287.95 (237.67~325.95)	19.47 (16.08~22.07)	− 0.39 (− 0.55~− 0.24)
**Eastern Europe**	125.38 (103.21~134.11)	54.04 (44.37~58.01)	100.72 (93.99~107.63)	40.05 (36.99~43.33)	− 1.3 (− 1.44~− 1.16)
**Eastern sub-Saharan Africa**	62.39 (37.66~110.88)	35.26 (24.60~54.37)	151.95 (110.81~187.83)	43.83 (33.07~52.92)	0.72 (0.62~0.82)
**High-income Asia Pacific**	102.63 (98.18~106.74)	56.08 (53.16~58.77)	116.96 (109.06~124.24)	41.07 (37.61~44.39)	− 0.96 (− 1.13~− 0.80)
**High-income North America**	183.11 (79.19~188.19)	58.14 (56.87~59.63)	284.65 (273.72~296.64)	57.13 (54.81~59.92)	− 0.1 (− 0.29~0.08)
**North Africa and Middle East**	158.77 (103.98~269.53)	49.15 (32.55~77.32)	240.16 (200.29~292.56)	42.22 (35.14~51.02)	− 0.38 (− 0.49~− 0.26)
**Oceania**	3.52 (2.50~5.64)	57.85 (42.37~85.48)	7.00 (5.11~10.58)	58.37 (43.45~84.18)	0.15 (0.07~0.23)
**South Asia**	369.72 (235.28~688.01)	35.87 (25.05~59.11)	705.85 (611.78~813.39)	42.6 (37.00~48.84)	0.59 (0.51~0.66)
**Southeast Asia**	133.06 (89.81~238.42)	31.44 (22.75~52.67)	261.12 (222.66~306.94)	40.17 (34.35~47.00)	1.05 (0.93~1.16)
**Southern Latin America**	23.26 (21.47~25.14)	47.14 (43.50~50.96)	31.77 (28.93~34.56)	45.14 (41.04~49.30)	− 0.13 (− 0.24~− 0.01)
**Southern sub-Saharan Africa**	20.25 (14.92~23.42)	44.10 (32.64~51.71)	32.29 (23.32~38.31)	44.59 (32.55~52.50)	− 0.21 (− 0.55~0.14)
**Tropical Latin America**	76.82 (71.52~81.77)	52.92 (49.84~55.71)	113.41 (106.70~118.33)	50.65 (47.54~53.23)	− 0.12 (− 0.22~− 0.02)
**Western Europe**	254.91 (246.17~265.24)	54.79 (52.76~57.05)	365.39 (343.08~386.36)	54.91 (51.16~58.51)	0.13 (− 0.06~0.32)
**Western sub-Saharan Africa**	66.12 (41.01~101.06)	32.44 (22.56~43.65)	170.78 (117.14~208.56)	37.83 (26.76~46.50)	0.47 (0.34~0.60)

*DALY* disability-adjusted life years

### The correlation between SDI and AML’s incidence and mortality

**Fig. 4:**
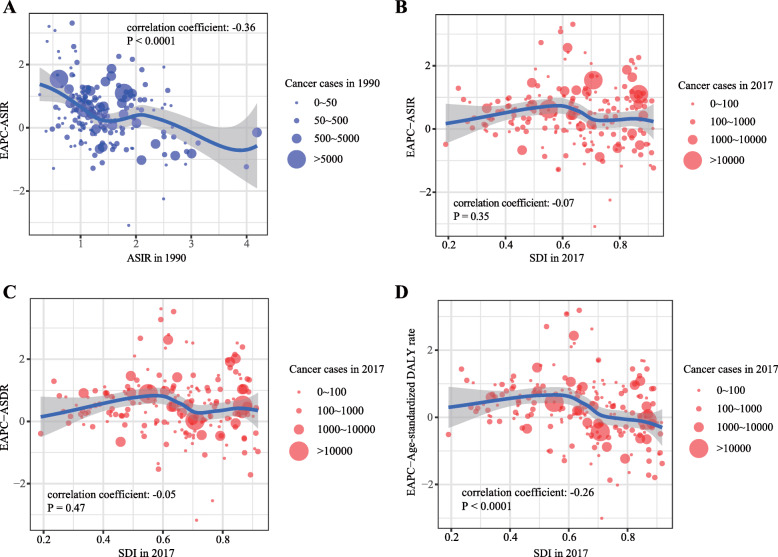
The correlation analyses of EAPCs-ASIR (1990) and EAPCs-SDI (2017). **a** The correlation between EAPC of ASIR and ASIR of 1990 in 195 countries or territories, **b** the correlation between EAPC of ASIR and SDI of 2017 in 195 countries or territories, **c** the correlation between EAPC of ASDR and SDI of 2017 in 195 countries or territories, and **d** the correlation between EAPC of age-standardized DALY rate and SDI of 2017 in 195 countries or territories. The size of circle represents the quantity of AML patients in one country or territory. Note: AML, acute myeloid leukemia; ASIR, age-standardized incidence rate; ASDR, age-standardized death rate; DALY, disability-adjusted life year; EAPC, estimated annual percentage change; SDI, socio-demographic index

**Fig. 5:**
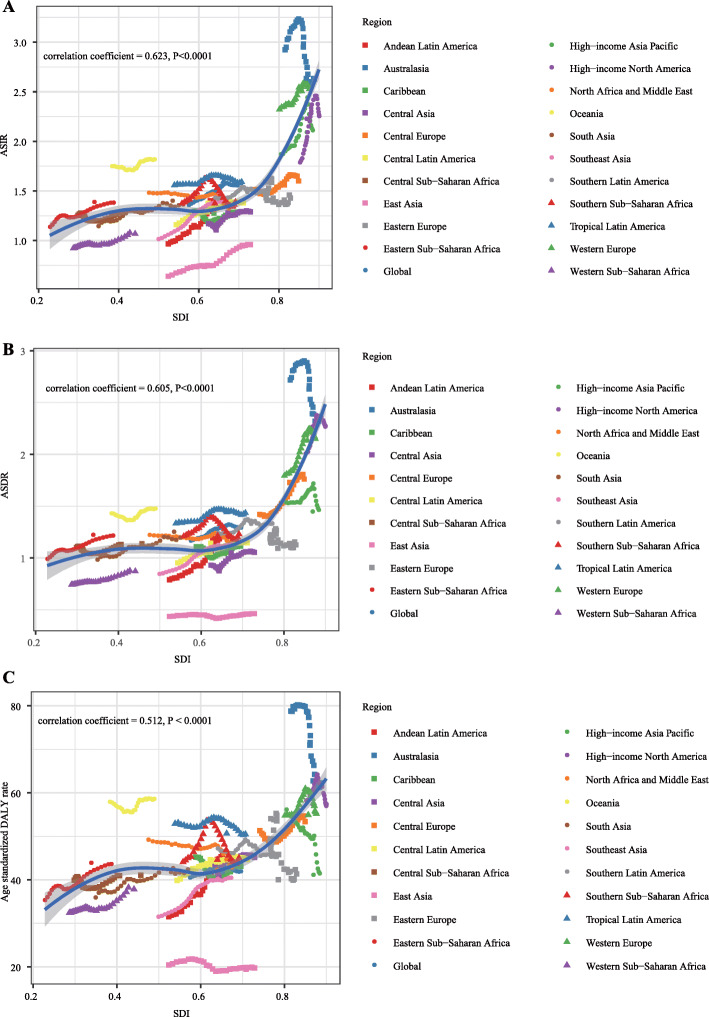
The change trends and correlation analyses of ASRs and SDI from 1990 to 2017. **a** The change trends and correlation of ASIR and SDI from 1990 to 2017 in 21 regions. **b** The change trends and correlation of ASDR and SDI from 1990 to 2017 in 21 regions. **c** The change trends and correlation of age-standardized DALY rate and SDI from 1990 to 2017 in 21 regions. Note: AML, acute myeloid leukemia; ASIR, age-standardized incidence rate; ASDR, age-standardized death rate; DALY, disability-adjusted life year; EAPC, estimated annual percentage change; SDI, socio-demographic index

Firstly, we calculated the correlation coefficient between ASIR in 1990 and the corresponding EAPC value. We found that the EAPC of ASIR was negatively correlated with ASIR in 1990 (correlation coefficient = − 0.36, *P* < 0.0001) which indicated that the incidence might be underestimated in regions with a low incidence rate (Fig. 4a). By calculating Pearson’s correlation coefficient, we assessed the correlation between SDI in 2017 and EAPC values of ASIR, ASDR, and age-standardized DALY rate in 195 countries. The results showed that the correlations between SDI and EAPCs of ASIR/ASDR were not statistically significant (Fig. 4b, c). However, the EAPC of age-standardized DALY rate was remarkably negatively correlated with SDI (Fig. 4d). Then, we investigate the correlation between SDI and ASIR, ASDR, and age-standardized DALY rate in 21 regions around the globe. The results showed that all ASRs values were markedly positively correlated with SDI (correlation coefficient of ASIR = 0.623, of ASDR = 0.605, of age-standardized DALY rate = 0.512, all *P* values < 0.0001) (Fig. 5a–c).

### The AML’s incidence and age structure

**Fig. 6:**
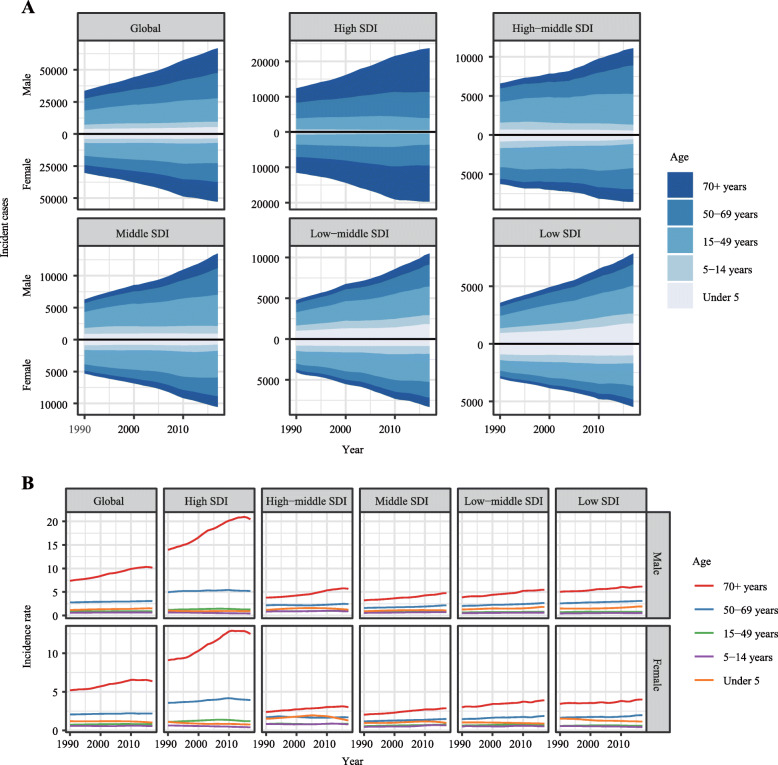
The incidence cases and corresponding ASIR of AML in different age groups from 1990 to 2017. **a** The incidence cases of AML in five different age groups in the globe and various regions. **b** The ASIR of AML in five different age groups in the globe and various regions. Note: AML, acute myeloid leukemia; ASIR, age-standardized incidence rate; SDI, socio-demographic index

We analyzed the incidence and its rate in five different age groups: under 5 years, 5~14 years, 15~49 years, 50~69 years, and above 70 years in the globe and different regions. The results demonstrated that most incidences were aged 50 years or older in the globe. Besides, in the high SDI region, patients aged 50 years or older accounted for approximately 80% AML’s incidence cases in 2017. In the low SDI regions, this ratio of patients aged 50 years or older in total incidences was about 35% (Fig. 6a). In all age groups, the incidence rate of patients aged 70 years or older was highest especially in the high SDI region (Fig. 6b).

### The AML-related mortality attributable risk factors

**Fig. 7:**
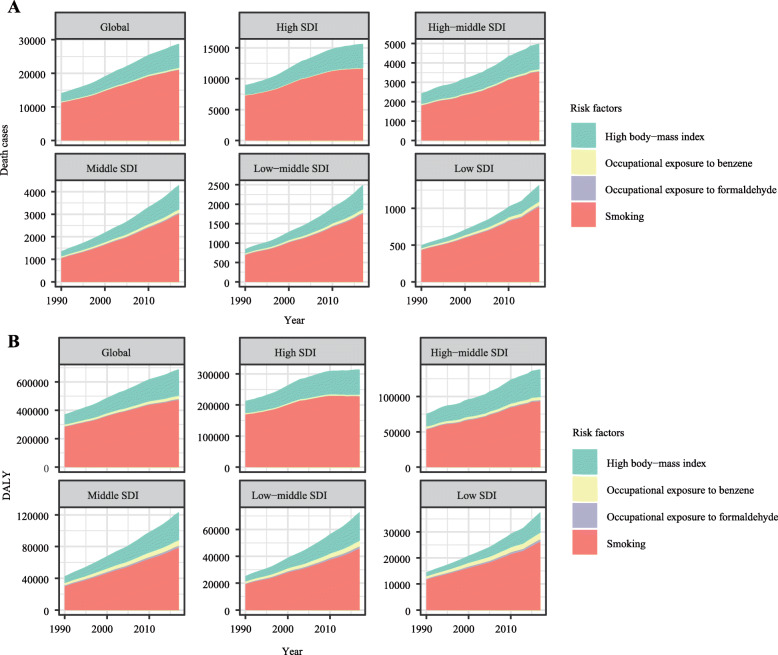
Risk factors contributing to AML-related death and DALY. **a** The four risk factors contributing to AML-related death from 1990 to 2017 in the globe and different regions; **b** the four risk factors contributing to AML-related DALY from 1990 to 2017 in the globe and different regions; Note: AML, acute myeloid leukemia; DALY, disability-adjusted life year; SDI, socio-demographic index

We searched the GBD database for the potential AML-related mortality attributable risk factors. Eventually, we found four risk factors contributing to AML-related death and DALY: high body mass index, occupational exposure to benzene, occupational exposure to formaldehyde, and smoking. Among all risk factors, smoking was the greatest contributor to AML-related death and DALY from 1990 to 2017 in the globe (Fig. 7a, b). Smoking was the second leading risk factor, and its contribution ratio was rapidly increased during the 28 years (Additional file 1: Figs. S1 and S2). For regions with different SDI values, it was observed that the influence of occupation exposure to carcinogens in the low SDI region was significantly higher than in the high SDI region.

## Discussion

In this study, we reported the incidence, death, and DALY data of AML based on GBD database. Besides, we analyzed the epidemiological trends of AML by calculating the EAPC values during the past 28 years. Generally, the AML’s incidence and related death were gradually increased in the globe. We found the incidence rate of AML was significantly higher in high SDI countries such as the UK which could be attributed to better cancer diagnosis and registry system, as well as population aging in these countries. Actually, aging is an important factor contributing to leukemogenesis. Accompanied by aging, multiple cancer-associated events occur including genomic alterations, protein homeostasis dysregulation, and mitochondrial dysfunction [33]. As an essential step of myeloid leukemogenesis, pre-leukemic clone acquires other co-operating mutations and develops into a new sub-clone which could outgrow normal hemopoietic stem cells at a rate boosted by some specific mutations [34]. A growing body of evidence indicated that these AML-related mutation-derived founder clones actually exist in healthy people but become more common with age. This phenomenon is termed age-related clonal hemopoiesis which is the precursor of AML and derived by alterations in some genes including *TET2*, *JAK2*, and *ASXL1* [34–36]. As a result, AML is mainly diagnosed in elder patients and rarely occurs before the age of 40 years [37]. Moreover, the prognosis of elder AML patients is poorer than younger patients which is associated with worse performance status at diagnosis, lower complete remission rate, and higher early death rate after intensive chemotherapy, as well as a higher risk of secondary AML [38]. With the aging of population in the globe, it is necessary to pay attention to the rapid increase of AML.

In addition to age, sociodemographic factors are also important variables in AML’s epidemiology especially for AML-related mortality. Consistent with the data of solid tumors, marital status is closely associated to the risk of AML-related death. Compared with unmarried AML patients, married or cohabitating counterparts had a lower risk of death [27, 39]. In terms of socioeconomic status, the prognosis of AML’s patients with low-income or residing in poverty-stricken regions was relatively poorer [3, 40]. Besides, multiple studies reported that race is a vital factor affecting the mortality rate of AML. The data from the SEER database showed that Pacific Islanders/Alaskan natives had the highest age-adjusted 5-year survival rate (16.7%), followed by Caucasians (16.4%), and African-Americans (12.1%). The relatively lower survival rate in AML patients from minority groups was also observed in other independent studies. These registry-based study in the USA demonstrated that the risk of AML-related mortality was significantly higher in African-Americans than Caucasians [40, 41]. Interventions such as providing earlier diagnosis and improving overall survival are necessary to address the disparity.

Apart from the epidemiological trend of AML, we also investigated the potential risk factors contributing to AML-related mortality. In this study, we found smoking was the primary risk factor for AML-related death and DALY. In multiple previous case-control studies, smoking was closely related with the increased risk of AML [42, 43]. A meta-analysis showed that both current and ever smokers had a higher risk to develop AML than non-smokers [44]. Tobacco smoke contains over 3800 chemicals and part of chemicals are potential carcinogens. It has been proved that tobacco smoke could increase the possibility of micronuclei formation and chromatid exchange in myeloid tissues [45]. Besides, smoking also is also positively associated with shorter remission and survival time, as well as higher pulmonary infections during AML treatment [46]. As the second leading risk factor behind smoking in this study, high body mass index has also been reported to herald the high risk of AML. Obesity before diagnosis was associated with AML in males and females [47]. A previous systematic review further proved that overweight or obesity was an unfavorable prognosis predictor for partial AML subtypes such as acute promyelocytic leukemia [48]. Benzene is an obsolete of chemotherapy agents which is a well-established risk factor for AML [49–51]. In spite of decades of environmental governance, the risk of exposure to this ubiquitous chemical still exists [49]. Similar to benzene, exposure to formaldehyde could also induce leukemia-related cytogenetic changes in myeloid progenitor cells [52]. Some cross-sectional studies of workers exposed to formaldehyde at factories using or producing formaldehyde showed that formaldehyde exposure damaged hematopoietic cells [53]. Notably, the risk of exposure to carcinogens including benzene in the low SDI region was significantly higher than in the high SDI region. For the low SDI region, there is still a long way to go to reduce the hazard of occupational exposure to carcinogens especially in the petrochemical industry and its related fields.

During the last few decades, the standard induction treatment for AML has always been based on 7 + 3 chemotherapy regimen. Generally, the complete remission rate is approximately 60–80% in younger patients and 40–60% in elder patients [54]. Post-remission regimens contain conventional chemotherapy or allogeneic hematopoietic stem cell transplantation [55]. However, for the last 2 or 3 years, a plethora of agents which target some specific mutations or cell survival signal pathways have been approved by FDA for AML treatment including FLT3 inhibitor, IDH2/IDH1 inhibitor, and BCL-2 inhibitor [20, 56]. Besides, some novel treatment strategies such as CD3-CD33 bispecific antibody [57], CD33-directed, or anti-CD123 CAR-T cells [58, 59] and immune checkpoint inhibitors are still in clinical trials [60]. These novel therapies, or in combination with conventional chemotherapy, will drastically change the landscape of AML treatment.

## Conclusion

Globally, the incidence rate and mortality rate of AML were gradually increased. Males and elder people had a higher risk to develop AML. The incidence rate of AML was positively correlated to SDI values which meant the incidence rate in the developed region was significantly higher than in the developing region. In the meanwhile, the incidence rate in some developing areas such as the middle SDI and low-middle SDI countries increased rapidly. Smoking, high body mass index, occupational exposure to benzene, and formaldehyde were mainly risk factors contributing to AML-related mortality. There is plenty of room to control occupational exposure to carcinogens especially in developing countries. Generally, considering the accelerated aging trend in the globe, the incidence rate and mortality rate of AML might further increase. Therefore, the policy-marker should rationally allocate public health resources to relieve the mushrooming burden of AML.

## Supplementary information

**Additional file 1: Table S1.** Top 20 countries or territories with most incidence cases in 1990. **Table S2.** Top 20 countries or territories with most death cases in 1990. **Table S3.** Top 20 countries or territories with highest DALY in 1990. **Table S4.** Top 20 countries or territories with highest ASIR in 1990. **Table S5.** Top 20 countries or territories with highest ASDR in 1990. **Table S6.** Top 20 countries or territories with highest age-standardized DALY rate in 1990. **Table S7.** Top 20 countries or territories with most incidence cases in 2017. **Table S8.** Top 20 countries or territories with most death cases in 2017. **Table S9.** Top 20 countries or territories with highest DALY in 2017. **Table S10.** Top 20 countries or territories with highest ASIR in 2017. **Table S11.** Top 20 countries or territories with highest ASDR in 2017. **Table S12.** Top 20 countries or territories with highest age-standardized DALY rate in 2017. **Table S13.** Top 10 countries or territories with the most rapid increase in ASIR. **Table S14.** Top 10 countries or territories with the most rapid increase in ASDR. **Table S15.** Top 10 countries or territories with the most rapid increase in age-standardized DALY rate. **Figure S1.** The contribution ratio of four risk factor for AML-related death from 1990 to 2017 in the globe and different regions. **Figure S2.** The contribution ratio of four risk factor for AML-related DALY from 1990 to 2017 in the globe and different regions.

## Data Availability

The datasets generated during and/or analyzed during the current study are available from the Global Health Data Exchange query tool (http://ghdx.healthdata.org/gbd-results-tool).

## Abbreviations

AML: Acute myeloid leukemia
GBD: Global burden disease
DALY: Disability-adjusted life year
SDI: Socio-demographic index
ASIR: Age-standardized incidence rate
ASDR: Age-standardized death rate
EAPC: Estimated annual percentage change
ASR: Age-standardized rate
