# Supplementary material for: The global burden and attributable risk factor analysis of acute myeloid leukemia in 195 countries and territories from 1990 to 2017: estimates based on the global burden of disease study 2017
Source: J Hematol Oncol. 2020 Jun 8;13:72. doi: 10.1186/s13045-020-00908-z (PMC7282046; doi:10.1186/s13045-020-00908-z)

**The global burden and attributable risk factor analysis of acute myeloid leukemia in 195 countries and territories from 1990 to 2017: estimates based on Global Burden of Disease Study 2017**

Ming Yi^1^, Anping Li^2^, Linghui Zhou^3^, Qian Chu^1^, Yongping Song^2^, Kongming Wu^1,2^*

1. Department of Oncology, Tongji Hospital of Tongji Medical College, Huazhong University of Science and Technology, Wuhan, 430030, China.
2. The Affiliated Cancer Hospital of Zhengzhou University & Henan Cancer Hospital, Zhengzhou, 450008, China.
3. Department of Oncology, The Second Affiliated Hospital of Xi’an Jiaotong University, Xi’an, 710004, China.

*** Co-corresponding author**

Kongming Wu, Department of Oncology, Tongji Hospital of Tongji Medical College, Huazhong University of Science and Technology, Wuhan, 430030, China. E-mail: wukm_lab@163.com

**Author emails:**

Ming Yi: 1978135000@qq.com;

Anping Li: li_anping@yahoo.com;

Linghui Zhou: 2544928065@qq.com;

Qian Chu: qianchu@tjh.tjmu.edu.cn;

Yongping Song:songyongping001@126.com;

Kongming Wu: wukm_lab@163.com.

**Supplementary Table 1:** Top 20 countries or territories with most incidence cases in 1990.

**Supplementary Table 2:** Top 20 countries or territories with most death cases in 1990.

**Supplementary Table 3:** Top 20 countries or territories with highest DALY in 1990.

**Supplementary Table 4:** Top 20 countries or territories with highest ASIR in 1990.

**Supplementary Table 5:** Top 20 countries or territories with highest ASDR in 1990.

**Supplementary Table 6:** Top 20 countries or territories with highest Age standardized DALY rate in 1990.

**Supplementary Table 7:** Top 20 countries or territories with most incidence cases in 2017.

**Supplementary Table 8:** Top 20 countries or territories with most death cases in 2017.

**Supplementary Table 9:** Top 20 countries or territories with highest DALY in 2017.

**Supplementary Table 10:** Top 20 countries or territories with highest ASIR in 2017.

**Supplementary Table 11:** Top 20 countries or territories with highest ASDR in 2017.

**Supplementary Table 12:** Top 20 countries or territories with highest Age standardized DALY rate in 2017.

**Supplementary Table 13:** Top 10 countries or territories with the most rapid increase in ASIR.

**Supplementary Table 14:** Top 10 countries or territories with the most rapid increase in ASDR.

**Supplementary Table 15:** Top 10 countries or territories with the most rapid increase in Age standardized DALY rate.

**Supplementary Figure 1:** The contribution ratio of four risk factor for AML-related death from 1990 to 2017 in the globe and different regions.

**Supplementary Figure 2:** The contribution ratio of four risk factor for AML-related DALY from 1990 to 2017 in the globe and different regions.

| Location | Incidence case |
| --- | --- |
| India | 7429.711 |
| China | 6931.975 |
| United States | 5395.239 |
| United Kingdom | 3410.366 |
| Japan | 2918.735 |
| Germany | 2665.869 |
| Russian Federation | 2213.948 |
| Brazil | 2009.794 |
| Turkey | 1376.937 |
| Pakistan | 1310.49 |
| France | 1289.947 |
| Indonesia | 1229.466 |
| Italy | 1156.014 |
| Bangladesh | 1146.392 |
| Ukraine | 1121.166 |
| Iran | 1021.872 |
| Mexico | 882.0161 |
| Spain | 739.5503 |
| Nigeria | 661.2332 |
| Poland | 596.6711 |

**Table 1: Top 20 countries or territories with most incidence cases in 1990.**

**Table 2: Top 20 countries or territories with most death cases in 1990.**

| Location | Deaths |
| --- | --- |
| United States | 6424.054 |
| India | 5675.327 |
| China | 4471.479 |
| Japan | 2527.871 |
| United Kingdom | 2522.677 |
| Germany | 2195.919 |
| Russian Federation | 1842.804 |
| Brazil | 1580.885 |
| France | 1238.761 |
| Italy | 1078.989 |
| Turkey | 1037.711 |
| Pakistan | 959.7782 |
| Ukraine | 903.7046 |
| Indonesia | 882.551 |
| Bangladesh | 829.5262 |
| Iran | 729.1683 |
| Mexico | 650.2986 |
| Poland | 616.7345 |
| Canada | 559.1212 |
| Australia | 535.8691 |

**Table 3: Top 20 countries or territories with highest DALY in 1990.**

| Location | DALY |
| --- | --- |
| India | 271597.9 |
| China | 235204.2 |
| United States | 167186.2 |
| Japan | 79564.15 |
| Brazil | 75686.03 |
| Russian Federation | 73481.43 |
| United Kingdom | 59359.85 |
| Germany | 58685.95 |
| Turkey | 50736.45 |
| Pakistan | 48169.23 |
| Indonesia | 44643.99 |
| Bangladesh | 43595.07 |
| Iran | 41672.06 |
| Ukraine | 38284.13 |
| Mexico | 34261.61 |
| France | 31892.56 |
| Italy | 29680.58 |
| Nigeria | 26245.49 |
| Ethiopia | 22063.99 |
| Poland | 21152.31 |

**Table 4: Top 20 countries or territories with highest ASIR in 1990.**

| Location | ASIR |
| --- | --- |
| United Kingdom | 4.187381 |
| Denmark | 3.995908 |
| Finland | 3.123485 |
| Australia | 3.006594 |
| Norway | 2.916213 |
| Iceland | 2.897186 |
| Andorra | 2.891112 |
| Turkey | 2.709877 |
| Israel | 2.683825 |
| Luxembourg | 2.657553 |
| Fiji | 2.633982 |
| Slovakia | 2.575273 |
| Bermuda | 2.546314 |
| Vanuatu | 2.522834 |
| Syria | 2.517605 |
| New Zealand | 2.507837 |
| Brunei | 2.504647 |
| Sweden | 2.50156 |
| Qatar | 2.499691 |
| Estonia | 2.475897 |

**Table 5: Top 20 countries or territories with highest ASDR in 1990.**

| Location | ASDR |
| --- | --- |
| Denmark | 3.157115 |
| United Kingdom | 2.872061 |
| Australia | 2.741419 |
| Israel | 2.681465 |
| New Zealand | 2.606073 |
| Finland | 2.455553 |
| Andorra | 2.446631 |
| Iceland | 2.426477 |
| Qatar | 2.388617 |
| Brunei | 2.346359 |
| Fiji | 2.29426 |
| Turkey | 2.216313 |
| Luxembourg | 2.192299 |
| Malta | 2.183264 |
| Bermuda | 2.181867 |
| Syria | 2.145318 |
| Norway | 2.140207 |
| Vanuatu | 2.116967 |
| Estonia | 2.054263 |
| United States | 2.027865 |

**Table 6: Top 20 countries or territories with highest Age standardized DALY rate in 1990.**

| Location | Age standardized DALY |
| --- | --- |
| Turkey | 91.0734 |
| Bermuda | 90.90802 |
| Fiji | 89.25369 |
| Denmark | 87.93966 |
| Vanuatu | 85.52866 |
| Brunei | 80.88324 |
| United Kingdom | 79.73414 |
| Australia | 79.40766 |
| Israel | 79.0932 |
| Syria | 78.50862 |
| Guam | 76.78716 |
| New Zealand | 75.51251 |
| Qatar | 74.30715 |
| Ukraine | 73.55599 |
| Barbados | 72.77957 |
| Iran | 72.59164 |
| Estonia | 71.71399 |
| Iceland | 70.54673 |
| Finland | 69.91348 |
| Virgin Islands, U.S. | 69.36944 |

**Table 7: Top 20 countries or territories with most incidence cases in 2017.**

| Location | Year | Incidence case |
| --- | --- | --- |
| India | 2017 | 15780.23 |
| China | 2017 | 13211.36 |
| USA | 2017 | 10589.61 |
| Japan | 2017 | 6590.896 |
| UK | 2017 | 4646.605 |
| Pakistan | 2017 | 3665.715 |
| Germany | 2017 | 3620.653 |
| Brazil | 2017 | 3497.131 |
| Italy | 2017 | 2718.771 |
| Indonesia | 2017 | 2581.362 |
| France | 2017 | 2408.41 |
| Russia | 2017 | 2122.545 |
| Spain | 2017 | 2061.47 |
| Nigeria | 2017 | 1849.129 |
| Turkey | 2017 | 1723.543 |
| Mexico | 2017 | 1674.712 |
| Bangladesh | 2017 | 1665.009 |
| Philippines | 2017 | 1557.131 |
| Thailand | 2017 | 1464.912 |
| Vietnam | 2017 | 1353.655 |

**Table 8: Top 20 countries or territories with most death cases in 2017.**

| Location | Year | Death cases |
| --- | --- | --- |
| India | 2017 | 13794.48 |
| USA | 2017 | 12005.09 |
| China | 2017 | 7066.33 |
| Japan | 2017 | 5157.226 |
| Germany | 2017 | 4012.838 |
| UK | 2017 | 3487.659 |
| Brazil | 2017 | 3198.642 |
| Italy | 2017 | 2718.1 |
| Pakistan | 2017 | 2625.11 |
| France | 2017 | 2478.22 |
| Indonesia | 2017 | 2033.271 |
| Russia | 2017 | 1938.231 |
| Turkey | 2017 | 1589.017 |
| Spain | 2017 | 1385.957 |
| Bangladesh | 2017 | 1353.566 |
| Mexico | 2017 | 1342.931 |
| Nigeria | 2017 | 1282.189 |
| Poland | 2017 | 1235.13 |
| Canada | 2017 | 1201.777 |
| Philippines | 2017 | 1125.779 |

**Table 9: Top 20 countries or territories with highest DALY in 2017.**

| Location | Year | DALY |
| --- | --- | --- |
| India | 2017 | 500565.5 |
| China | 2017 | 261691 |
| USA | 2017 | 258305.8 |
| Pakistan | 2017 | 134839.2 |
| Brazil | 2017 | 110484 |
| Japan | 2017 | 97783.35 |
| Germany | 2017 | 80640.46 |
| Indonesia | 2017 | 80157.65 |
| Nigeria | 2017 | 76680.67 |
| UK | 2017 | 68264.52 |
| Russia | 2017 | 60353.22 |
| Bangladesh | 2017 | 59351.07 |
| Turkey | 2017 | 56929 |
| Mexico | 2017 | 55408.99 |
| Philippines | 2017 | 54470.28 |
| Italy | 2017 | 53305.46 |
| France | 2017 | 47751.15 |
| Ethiopia | 2017 | 45451.18 |
| Iran | 2017 | 43550.67 |
| Vietnam | 2017 | 37399.13 |

**Table 10: Top 20 countries or territories with highest ASIR in 2017.**

| Location | Year | ASIR |
| --- | --- | --- |
| UK | 2017 | 4.054558 |
| Brunei | 2017 | 3.427758 |
| Slovakia | 2017 | 3.141717 |
| Vanuatu | 2017 | 3.071483 |
| Honduras | 2017 | 3.057412 |
| Fiji | 2017 | 3.017329 |
| Denmark | 2017 | 2.988433 |
| Austria | 2017 | 2.86525 |
| Lithuania | 2017 | 2.85219 |
| Finland | 2017 | 2.820226 |
| Malta | 2017 | 2.808917 |
| Estonia | 2017 | 2.808294 |
| Luxembourg | 2017 | 2.759047 |
| Australia | 2017 | 2.692129 |
| Switzerland | 2017 | 2.642054 |
| Netherlands | 2017 | 2.636667 |
| Andorra | 2017 | 2.621478 |
| Spain | 2017 | 2.582783 |
| Guam | 2017 | 2.491232 |
| Northern Mariana Islands | 2017 | 2.466887 |

**Table 11: Top 20 countries or territories with highest ASDR in 2017.**

| Location | Year | ASDR |
| --- | --- | --- |
| Brunei | 2017 | 2.914647 |
| UK | 2017 | 2.844505 |
| Vanuatu | 2017 | 2.628667 |
| Israel | 2017 | 2.616937 |
| Malta | 2017 | 2.608995 |
| Andorra | 2017 | 2.571172 |
| Fiji | 2017 | 2.544999 |
| Denmark | 2017 | 2.484842 |
| Luxembourg | 2017 | 2.475172 |
| New Zealand | 2017 | 2.456655 |
| Honduras | 2017 | 2.416783 |
| Australia | 2017 | 2.380824 |
| Lithuania | 2017 | 2.366989 |
| Austria | 2017 | 2.323829 |
| USA | 2017 | 2.305626 |
| Germany | 2017 | 2.281694 |
| Greece | 2017 | 2.269509 |
| Cyprus | 2017 | 2.242385 |
| Belgium | 2017 | 2.109717 |
| Ireland | 2017 | 2.106498 |

**Table 12: Top 20 countries or territories with highest Age standardized DALY rate in 2017.**

| Location | Year | Age standardized DALY |
| --- | --- | --- |
| Brunei | 2017 | 104.9479 |
| Fiji | 2017 | 103.2245 |
| Vanuatu | 2017 | 100.8913 |
| Honduras | 2017 | 86.29899 |
| Barbados | 2017 | 75.84996 |
| Syria | 2017 | 75.57876 |
| Lithuania | 2017 | 73.46858 |
| Guam | 2017 | 71.12687 |
| Malta | 2017 | 69.07033 |
| Israel | 2017 | 69.03053 |
| Turkey | 2017 | 68.76129 |
| UK | 2017 | 68.4673 |
| Virgin Islands | 2017 | 67.64277 |
| Eritrea | 2017 | 67.45024 |
| Afghanistan | 2017 | 66.87802 |
| Pakistan | 2017 | 65.72527 |
| Andorra | 2017 | 65.53126 |
| New Zealand | 2017 | 65.51483 |
| Luxembourg | 2017 | 65.34095 |
| Bahamas | 2017 | 64.39691 |

| Country | EAPC-ASIR | Low limits | Upper limits |
| --- | --- | --- | --- |
| Ecuador | 3.312 | 2.937 | 3.689 |
| El Salvador | 3.208 | 2.591 | 3.828 |
| Dominican Republic | 3.079 | 2.803 | 3.357 |
| Guatemala | 2.733 | 2.438 | 3.028 |
| Philippines | 2.571 | 2.37 | 2.772 |
| Paraguay | 2.424 | 2.132 | 2.717 |
| Jamaica | 2.414 | 2.049 | 2.782 |
| Austria | 2.259 | 2.134 | 2.384 |
| Saint Vincent and the Grenadines | 2.208 | 1.807 | 2.611 |
| Lithuania | 2.166 | 1.773 | 2.56 |

**Table 13: Top 10 countries or territories with the most rapid increase in ASIR**

**Table 14: Top 10 countries or territories with the most rapid increase in ASDR**

| Country | EAPC-ASDR | Lower limits | Upper limits |
| --- | --- | --- | --- |
| El Salvador | 3.618 | 2.93 | 4.312 |
| Ecuador | 3.528 | 3.131 | 3.928 |
| Dominican Republic | 3.272 | 2.983 | 3.562 |
| Paraguay | 2.788 | 2.471 | 3.107 |
| Guatemala | 2.67 | 2.387 | 2.954 |
| Philippines | 2.624 | 2.453 | 2.794 |
| Jamaica | 2.555 | 2.152 | 2.961 |
| Georgia | 2.519 | 1.258 | 3.796 |
| Austria | 2.39 | 2.14 | 2.64 |
| Saint Vincent and the Grenadines | 2.355 | 1.965 | 2.748 |

**Table 15: Top 10 countries or territories with the most rapid increase in Age standardized DALY rate**

| Country | EAPC-DALY rate | Lower limits | Upper limits |
| --- | --- | --- | --- |
| Ecuador | 3.186 | 2.807 | 3.566 |
| El Salvador | 3.093 | 2.427 | 3.764 |
| Dominican Republic | 3.075 | 2.772 | 3.378 |
| Guatemala | 2.702 | 2.428 | 2.976 |
| Jamaica | 2.631 | 2.264 | 3 |
| Philippines | 2.43 | 2.192 | 2.669 |
| Paraguay | 2.204 | 1.887 | 2.521 |
| Saint Vincent and the Grenadines | 2.101 | 1.704 | 2.5 |
| Azerbaijan | 1.704 | 1.41 | 1.999 |
| Georgia | 1.688 | 0.467 | 2.924 |


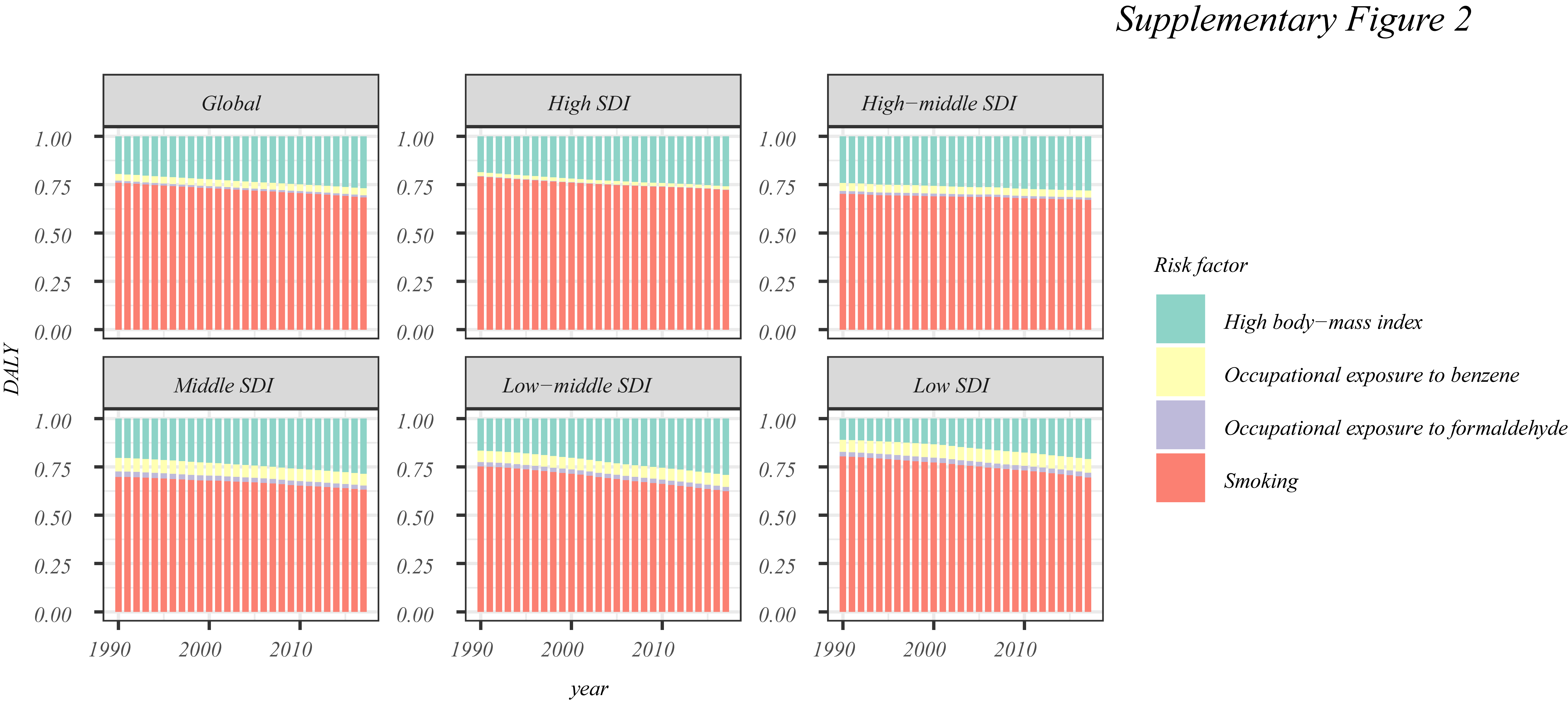

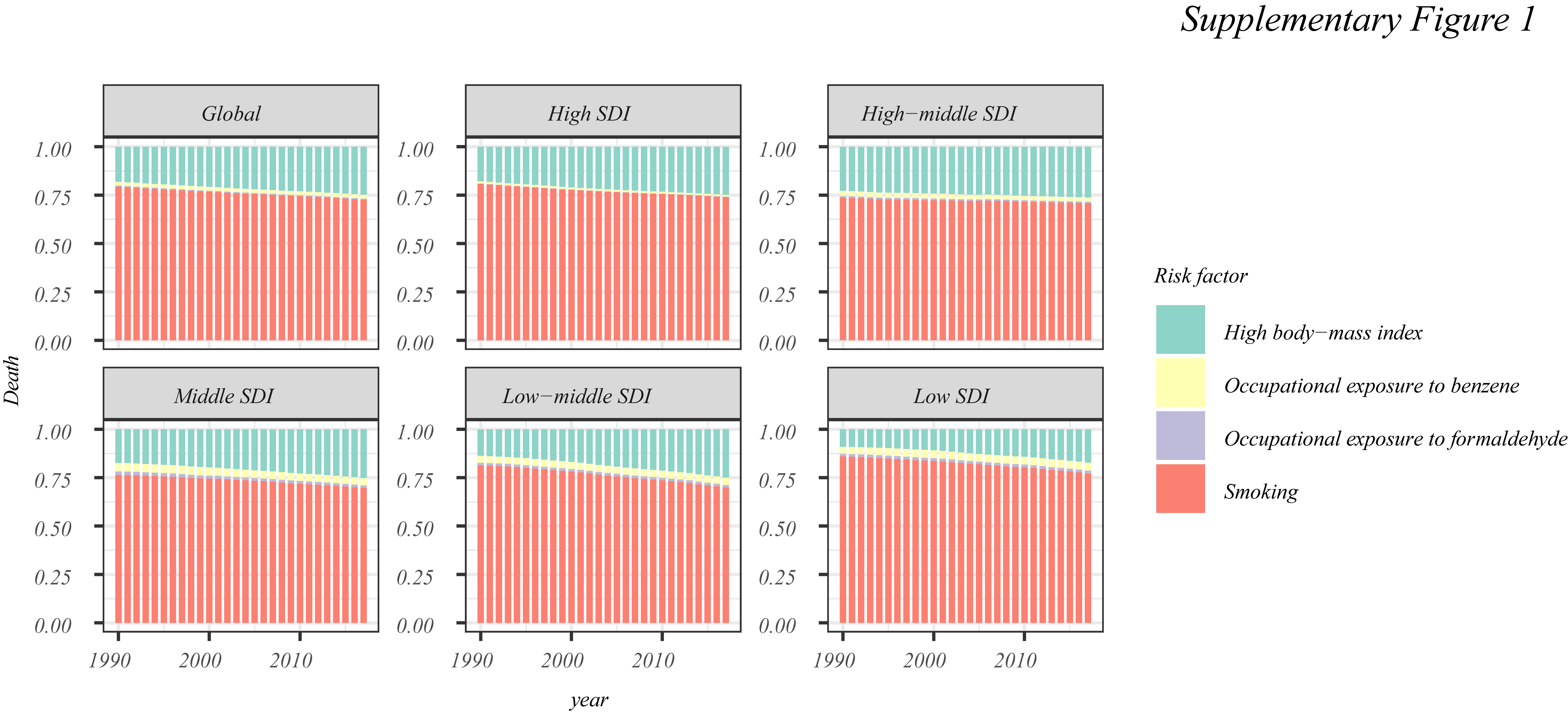

Supplement: Supplementary file 1 — Additional file 1: Table S1. Top 20 countries or territories with most incidence cases in 1990. Table S2. Top 20 countries or territories with most death cases in 1990. Table S3. Top 20 countries or territories with highest DALY in 1990. Table S4. Top 20 countries or territories with highest ASIR in 1990. Table S5. Top 20 countries or territories with highest ASDR in 1990. Table S6. Top 20 countries or territories with highest age-standardized DALY rate in 1990. Table S7. Top 20 countries or territories with most incidence cases in 2017. Table S8. Top 20 countries or territories with most death cases in 2017. Table S9. Top 20 countries or territories with highest DALY in 2017. Table S10. Top 20 countries or territories with highest ASIR in 2017. Table S11. Top 20 countries or territories with highest ASDR in 2017. Table S12. Top 20 countries or territories with highest age-standardized DALY rate in 2017. Table S13. Top 10 countries or territories with the most rapid increase in ASIR. Table S14. Top 10 countries or territories with the most rapid increase in ASDR. Table S15. Top 10 countries or territories with the most rapid increase in age-standardized DALY rate. Figure S1. The contribution ratio of four risk factor for AML-related death from 1990 to 2017 in the globe and different regions. Figure S2. The contribution ratio of four risk factor for AML-related DALY from 1990 to 2017 in the globe and different regions. [file 13045_2020_908_MOESM1_ESM.docx]
